# Utility of the neutrophil-to-lymphocyte ratio extends to patients from East Asia

**DOI:** 10.1172/JCI202712

**Published:** 2026-03-16

**Authors:** Seong-Keun Yoo, Jinha Hwang, Jung Yong Hong, Seung Tae Kim, Se Hoon Park, Joon Oh Park, Jeeyun Lee

**Affiliations:** 1 Samsung Precision Genome Medicine Institute, Samsung Medical Center, Seoul, South Korea.; 2 Division of Hematology-Oncology, Department of Medicine, Samsung Medical Center, Sungkyunkwan University School of Medicine, Seoul, South Korea.

**Keywords:** Clinical Research, Oncology, Biomarkers, Immunotherapy

**To the Editor:** Ang et al. found that the neutrophil-to-lymphocyte ratio (NLR) predicts immune checkpoint inhibitor (ICI) outcomes mainly in non-Hispanic white (NHW) patients with cancer, with limited association in non-Hispanic black (NHB), Asian, and Hispanic populations (1). This observation, that NLR appears to be an effective biomarker only in NHW patients, suggests that approaches leveraging blood-based markers (2) may exhibit substantially different predictive performance across racial and ethnic groups. Notably, a recent meta-analysis including 129 studies — including 83 from China, Japan, Korea, Spain, Turkey, and South Africa — encompassing 18,780 patients reported that elevated pretreatment NLR is consistently associated with poorer ICI outcomes across diverse regions and tumor types (3). However, the authors acknowledged important limitations, including the substantial between-study heterogeneity arising from variability in NLR cutoffs, follow-up durations, and frequent reliance on univariable rather than fully adjusted multivariable estimates.

To evaluate the utility of NLR as a biomarker for ICI outcomes in East Asian populations, we analyzed 3,173 patients across seven cancer types who received ICIs between 2014 and 2024 at Samsung Medical Center, Korea (Supplemental Figure 1 and Supplemental Table 1; supplemental material available online with this article; https://doi.org/10.1172/JCI202712DS1). Using the upper tertile definition of NLR-high, as in Ang et al., we confirmed that pretreatment NLR was significantly associated with overall survival (OS) in the pooled cohort and in tumor-specific subgroups (Figure 1A). Only 2 cancer types with limited sample sizes, sarcoma (*n* = 162) and colorectal cancer (*n* = 41), failed to show a significant association between pretreatment NLR and OS. Our observation remained significant after adjustment for multiple clinical characteristics (Figure 1B). Moreover, dose-dependent relationships between NLR cutoffs and OS were observed in the pooled cohort and in tumor-specific analyses, except for colorectal cancer (Figure 1C). We further evaluated the association between pretreatment NLR and duration of treatment as a surrogate for progression-free survival. Notably, statistical significance was maintained in this analysis among the cancer types where NLR was associated with OS (Figure 1D).

In the analysis by Ang et al., the failure to observe a significant association between NLR and ICI outcomes in racial groups other than NHW may be attributable to several factors. First, although Ang et al. performed power calculations, the lack of statistical significance in NHB, Asian, and Hispanic groups may still stem from limited effective sample sizes. Second, current racial categories used in U.S. clinical research often aggregate diverse ancestral and geographic origins into overly broad groups, such as “Asian,” which encompasses highly heterogeneous populations (e.g., East, South, and Southeast Asians). Such coarse categorization can lead to signal dilution, whereby a confirmed NLR-ICI association in East Asians may be attenuated when pooled with other Asian populations, if the association does not hold in those groups. As a result, clinically relevant associations may be overlooked, as previously highlighted (4).

In conclusion, our data show that NLR can serve as a biomarker for ICI outcome in East Asian populations, and that careful interpretation is warranted when generalizing the lack of an NLR-ICI association observed among U.S. NHB, Asian, and Hispanic populations to other countries and racial or ethnic groups.

## Funding Support

- This research was supported by a grant of the Korea Health Technology R&D Project through the Korea Health Industry Development Institute (KHIDI), funded by the Ministry of Health & Welfare, Republic of Korea (grant number: RS-2020-KH088685).

## Supplementary Material

Supplemental data

Supplemental table 2

## Figures and Tables

**Figure 1:**
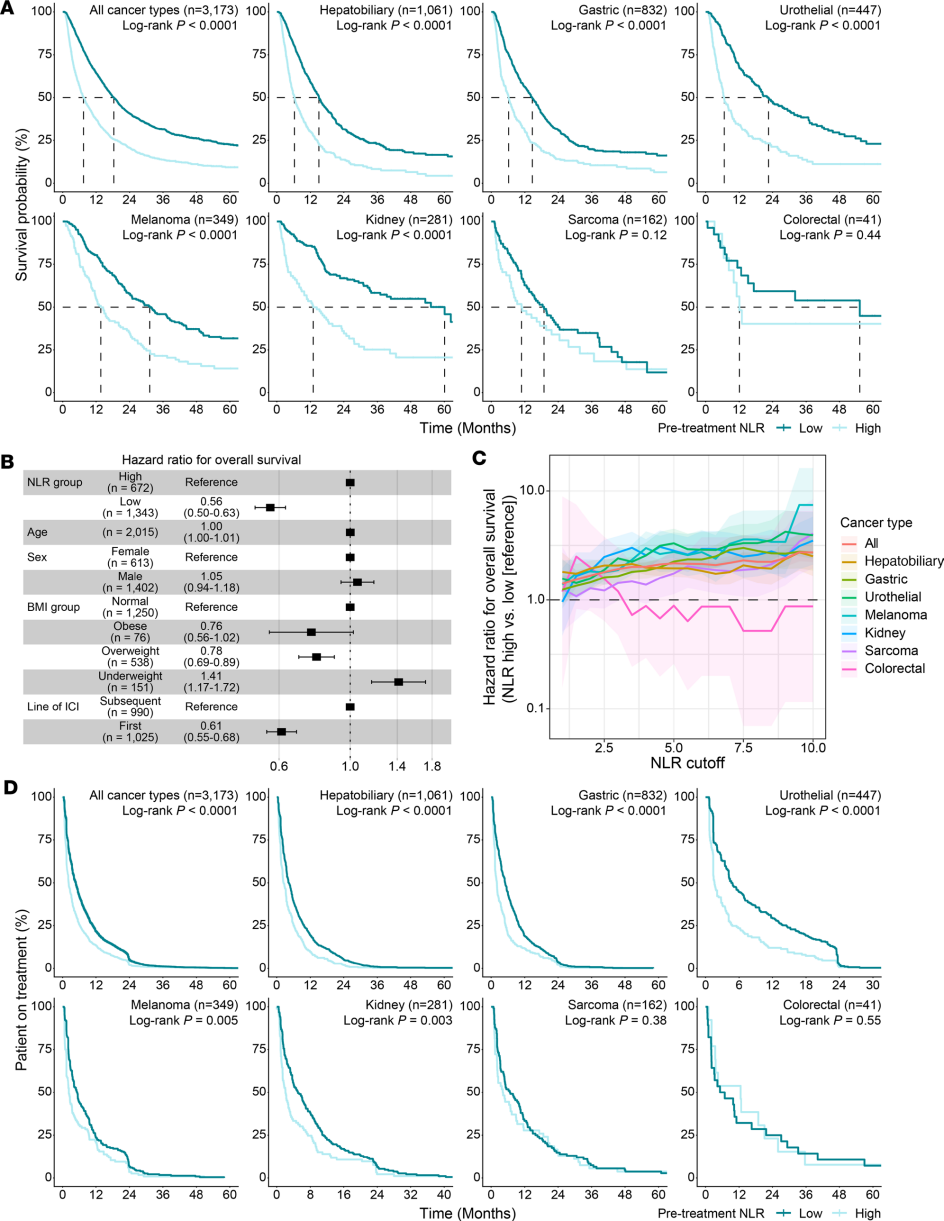
Utility of NLR in Korean patients. (**A**) Kaplan-Meier curves comparing OS between pretreatment NLR groups. *P* values are from 2-sided log-rank tests. (**B**) Forest plot of hazard ratios (HRs) for OS from a multivariable Cox proportional hazards model. BMI, body mass index. (**C**) HRs for OS across pretreatment NLR groups defined by various candidate cutoffs (1.0–10.0 in 0.5-unit increments). Solid lines indicate the estimated HRs; shaded bands represent the 95% CIs. (**D**) Kaplan-Meier curves comparing duration of treatment between pre-treatment NLR groups. *P* values are from 2-sided log-rank tests.

## Version 1 03/16/2026

Electronic publication
