# Supplementary material for: Utility of the neutrophil-to-lymphocyte ratio extends to patients from East Asia
Source: J Clin Invest. 2026 Mar 16;136(6):e202712. doi: 10.1172/JCI202712 (PMC12987652; doi:10.1172/JCI202712)
Supplement: Supplemental data [file jci-136-202712-s274.pdf]

MATERIALS AND METHODS

Patients

This study was approved by the Institutional Review Board (IRB) of Samsung Medical Center (IRB# 2021-09-052). In this retrospective study, we identified 3,565 patients with solid tumors who received at least two doses of immune checkpoint inhibitors (ICIs) in the palliative setting between 2014 and 2024 at Samsung Medical Center (**Figure S1**). Among these, 297 patients with inadequate laboratory data were excluded, leaving 3,268 patients with both pre-treatment and on-treatment neutrophil to lymphocyte ratio (NLR) measurements. We further excluded 95 patients with cancer types represented by fewer than 40 cases, resulting in a final analytic cohort of 3,173 patients. The median age was 62 years (interquartile range [IQR], 54-70), and 68.01% were male (n = 2,158; **Table S1**). Based on body mass index (BMI), 4.76% of patients were underweight, 39.39% were normal weight, 16.96% were overweight, and 2.40% were obese. BMI data were unavailable for 36.39% of the cohort. The median pre-treatment and on-treatment NLR values were 2.41 (IQR, 1.61-3.84) and 2.35 (IQR, 1.57-3.78), respectively. ICIs were given as first-line therapy in 48.38% of patients and as subsequent-line therapy in 51.62%. Cancer types were distributed as follows: hepatobiliary (33.44%), gastric (26.22%), urothelial (14.09%), melanoma (11.00%), kidney (8.86%), sarcoma (5.11%), and colorectal cancers (1.29%). The median overall survival for the entire cohort was 14.27 months (95% confidence interval, 13.51-15.06).

Clinical data

Data was extracted from the Clinical Data Warehouse DARWIN-C of Samsung Medical Center for this study. All pre-treatment clinical features, including NLR and BMI, were collected on the day of, or within 21 days before, the first ICI infusion. In cases where chemotherapy preceded ICI administration within a combination regimen (1), the date of the first ICI dose was defined as the baseline point. The NLR was calculated by dividing the absolute neutrophil count by the absolute lymphocyte count. In the pooled cohort, tertile cutoffs for NLR classification were defined using values derived from the entire study population, whereas in the cancer type-specific analyses, NLR tertiles were defined using cancer type-specific cutoffs. Detailed patient-level data are available in **Table S2**.

Outcome

Overall survival was defined as the time from the first infusion of the treatment regimen to death from any cause. Patients whose national health insurance coverage had expired were also considered deceased, with the date of death assigned as the day before the expiration date. Patients who were alive at the data cutoff were censored at the date of their last follow-up. Duration of treatment was calculated as the time interval from the date of the first cycle to the date of the last cycle.

Statistical analysis

All statistical analyses were performed in R (v4.5.0) using survival (v3.8-3), survminer (v0.5.1), and stats (v4.5.0) packages.

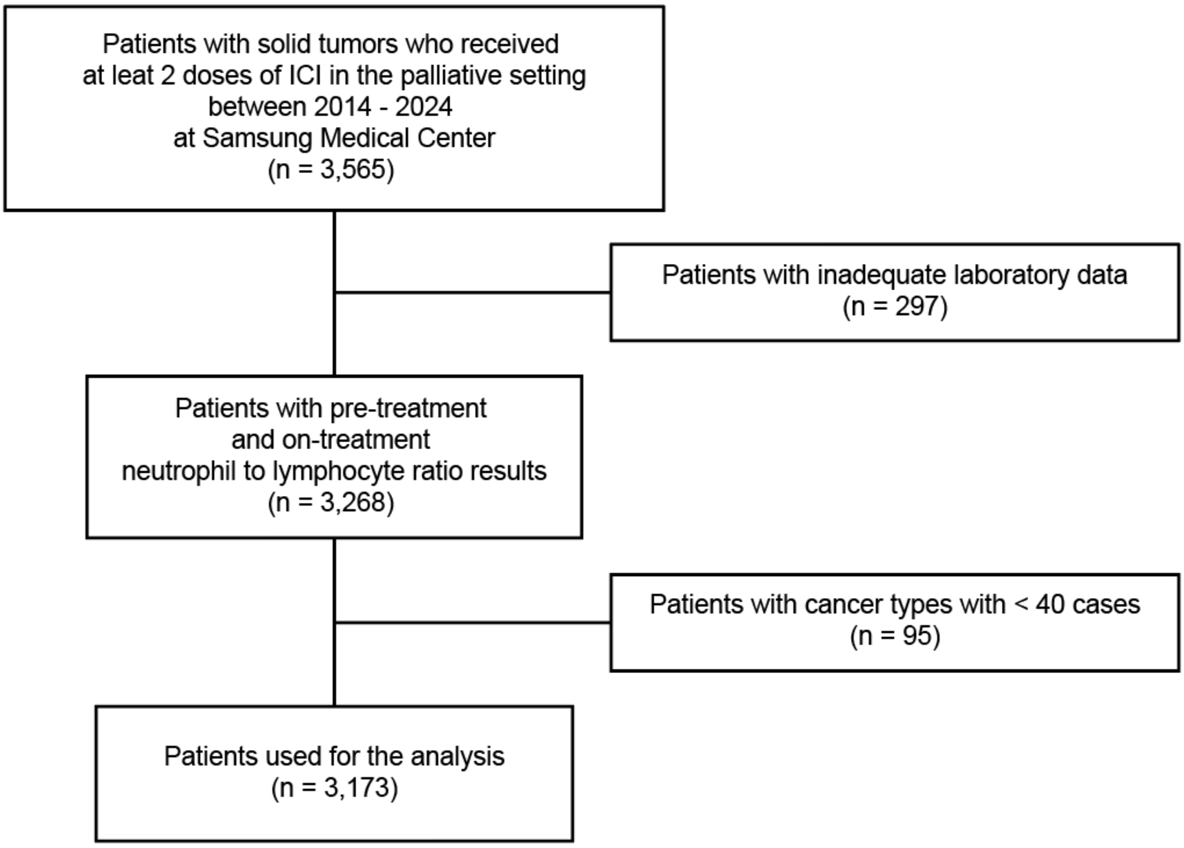

Figure S1. CONSORT diagram showing sample collection and filtering process.

Table S1. Patient characteristics.

Characteristics	Total patients (n=3,173)
**Sex, n (%)**	
Female	1,015 (31.99%)
Male	2,158 (68.01%)
**Age (years)**	
Median (IQR)	62 (54-70)
**BMI group, n (%)**	
Underweight	151 (4.76%)
Normal	1,250 (39.39%)
Overweight	538 (16.96%)
Obese	76 (2.40%)
Not available	1,158 (36.50%)
**Pre-treatment NLR**	
Median (IQR)	2.41 (1.61-3.84)
**ICI line of therapy, n (%)**	
First	1,535 (48.38%)
Subsequent	1,638 (51.62%)
**Cancer type, n (%)**	
Hepatobiliary	1061 (33.44%)
Gastric	832 (26.22%)
Urothelial	447 (14.09%)
Melanoma	349 (11.00%)
Kidney	281 (8.86%)
Sarcoma	162 (5.11%)
Colorectal	41 (1.29%)
**Overall survival**	
Median (95% CI)	14.27 (13.51-15.06)
**Duration of treatment**	
Median (95% CI)	3.68 (3.52-4.01)

IQR, interquartile range; BMI, body mass index; NLR, neutrophil to lymphocyte ratio; ICI, immune checkpoint inhibitor; CI, confidence interval

Reference

1. An M, Mehta A, Min BH, Heo YJ, Wright SJ, Parikh M, et al. Early Immune Remodeling Steers Clinical Response to First-Line Chemoimmunotherapy in Advanced Gastric Cancer. *Cancer Discov.* 2024;14(5):766-85.
